# Crystal structure of the lead-containing organic–inorganic hybrid: (C_18_H_26_N_2_)_3_[Pb_4_I_14_(DMSO)_2_]·2DMSO

**DOI:** 10.1107/S2056989018016584

**Published:** 2018-11-27

**Authors:** Li Li, Dan Zhao, Zhi Liu, Dingchao Zhang, Zhenhao Hu, Kunlun Li, Jing Yang

**Affiliations:** a State Key Laboratory of Crystal Materials, Shandong University, Jinan 250100, Shandong Province, People’s Republic of China; b School of Chemistry and Environmental Engineering, Shandong University of Science and Technology, Qingdao, People’s Republic of China

**Keywords:** organic-inorganic hybrid, crystal structure, hydrogen-bonding inter­actions, π–π inter­actions

## Abstract

The title compound, tris­(1,1′-dibutyl-4,4′-bi­pyridine-1,1′-diium) bis­(dimethyl sulfoxide)di-μ_3_-iodido-tetra-μ_2_-iodido-octa­iodido­tetra­lead(II) dimethyl sulfoxide di­solvate, (C_18_H_26_N_2_)_3_[Pb_4_I_14_(C_2_H_6_OS)_2_]·2C_2_H_6_OS, belongs to a class of organic–inorganic hybrid materials with novel functionalities. In this compound, C—H⋯O and C—H⋯I hydrogen-bonding inter­actions, π–π inter­actions, other short contacts and Pb octa­hedral chains are present, extending the crystal structure into a three-dimensional supra­molecular network.

## Abstract 2

The compound tris­(1,1′-dibutyl-4,4′-bi­pyridine-1,1′-diium) bis­(dimethyl sulfoxide)di-μ_3_-iodido-tetra-μ_2_-iodido-octa­iodido­tetra­lead(II) dimethyl sulfoxide tetra­solvate belongs a class of organic–inorganic hybrid materials with novel functionalities. In this compound, C—H⋯O and C—H⋯I hydrogen-bonding inter­actions, π–π inter­actions, other short contacts and Pb octa­hedral chains are present, extending the crystal structure into a three-dimensional supra­molecular network.

## Chemical context   

Organic–inorganic hybrid materials have attracted more and more attention from researchers because of their inter­esting physical properties and novel functionalities, such as magnetism, ferroelectricity, electrical/optical properties and photochromism (Yao *et al.*, 2017 ▸). The inorganic components provide rich structural possibilities, including discrete clusters, chains, layers and open frameworks, which dominate the significant electrical, optical and magnetic properties in hybrids (Sun *et al.*, 2018 ▸). The organic moieties may exhibit unique mol­ecular properties such as hyperpolarizability, photochromicity and polymerizability (Tang & Guloy, 1999 ▸). The title mol­ecule was prepared by the reaction of viologens (*N*,*N*′-disubstituted-4,4′-bipyridinium) and a metal halide. Viologens show excellent redox and chemical stability. In addition, they can act as effective templates for the construction of various organic–inorganic hybrids, charge-transfer complexes and supra­molecular systems (Liu *et al.*, 2017 ▸). As lead is a heavy *p*-block metal in the IVA group, lead(II) halide-based organic–inorganic hybrids possess a large radius, a flexible coordination environment, and variable stereochemical activities of the lead center (Li *et al.*, 2012 ▸).
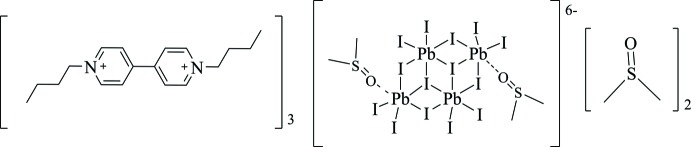


## Structural commentary   

**Figure 1:**
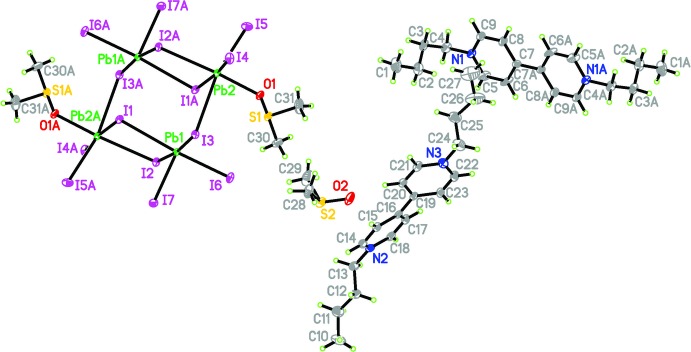
The mol­ecular structure of the title compound. Displacement ellipsoids are drawn at the 25% probability level. The second lattice DMSO molecule and the third VB cation, generated by symmetry, are omitted for clarity. Symmetry code: (A) −*x*, −*y*, −*z*.

The title compound crystallizes in the triclinic system in space group *P*ī. The asymmetric unit consists of half a [(Pb_4_I_14_)]^6−^ trianion, one and a half BV^2+^ (BV^2+^ = 1,1′-dibutyl-4,4′- bipyridinium) dications and two DMSO mol­ecules, as shown in Fig. 1 ▸. The BV^2+^ cation is located on a general position and adopts a non-planar structure, with a dihedral angle of 27.5 (3)° between the planes of the pyridinium rings. In the bipyridinium rings, C—N bond lengths vary from 1.335 (9) to 1.499 (10) Å and C—C bond lengths from 1.336 (17) to 1.636 (17) Å. C—N—C bond angles are in the range 118.6 (6)–121.1 (7)° and C—C—C bond angles in the range 107.9 (9)–122.1 (6)°. The inorganic anion can be considered as a set of mixed face-shared/edge-shared octa­hedra (Krautscheid *et al.*, 2001 ▸). Pb1—I bond lengths range from 3.0765 (5) to 3.4315 (5) Å and Pb2—I bond lengths from 3.0802 (5) to 3.4010 (5) Å. I—Pb1—I bond angles are in the range 82.007 (13)–172.112 (13)° and O—Pb2—I bond angles in the range 82.78 (10)-174.71 (9)°. All the above angles deviate from the angles of an ideal octa­hedron (90 and 180°) due to the stereochemical activity of the Pb (6*s* ^2^) lone pairs (Li *et al.*, 2005 ▸).

## Supra­molecular features   

**Figure 2:**
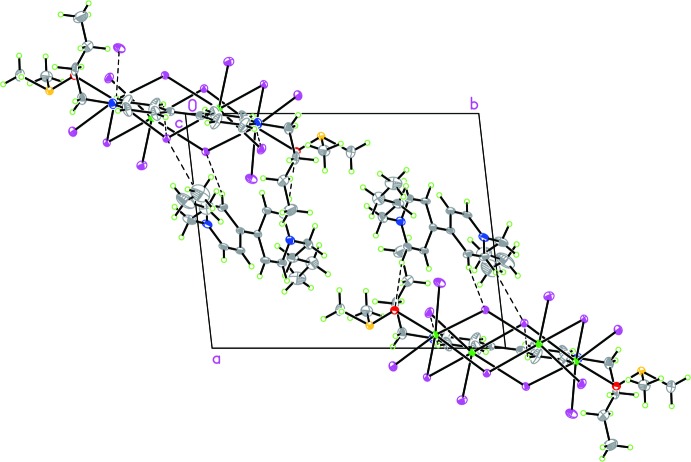
The crystal packing of the title compound with hydrogen bonds (Table 1 ▸) shown as dashed lines.

In the compound, the organic species inter­act with the inorganic [(Pb_4_I_14_)]^6−^ and DMSO via C—H⋯I and C—H⋯O hydrogen bonds (Table 1 ▸). The C⋯I distances are in the range 3.668 (8)–3.940 (10) Å while the C⋯O distances are 3.093 (9) and 3.517 (10) Å. The C—H⋯I angle values vary from 136 to 168°. Hydrogen bonds between the anionic entities [(Pb_4_I_14_)]^6−^ and organic species play an important role in stabilizing the crystal structure (Fig. 2 ▸). In addition, there are weak π–π inter­actions between adjacent free BV^2+^ cations with centroid-to-centroid distances between the pyridyl groups ranging from 4.249 (4) to 4.796 (4) Å (Table 2 ▸).

## Database survey   

Lead(II) iodide complexes have been reported whose structures include chains of face-sharing ideal PbI_6_ octa­hedra (Krautscheid *et al.*, 2001 ▸; She *et al.*, 2014 ▸) and chains of corner-sharing PbI_6_ octa­hedra (Wang *et al.*, 1995 ▸). The structure of 1,1′-dibutyl-4,4′-bipyridinium diiodide was reported by our research group (Zhao *et al.*, 2012 ▸). Typical Pb–I-based hybrids templated with alkyl viologen cations include, for example, [(Pb_6_I_22_)(DMF)_2_(DPB)_5_] (Zhang *et al.*, 2015 ▸), (C_21_H_27_N_3_)[Pb_3_I_9_] (Hong-Xu *et al.*, 2010 ▸), (C_14_H_18_N_2_)[Pb_2_I_6_] (Pradeesh *et al.*, 2010 ▸) and [IV][Pb_2_I_6_] (Kim *et al.*, 2018 ▸).

## Synthesis and crystallization   

NaI (0.23 g, 1.5 mmol), PbI_2_ (0.46 g, 1.0 mmol) and 10 ml of methanol were stirred under an argon atmosphere until dissolved. 1,1′-Dibutyl-4,4′-bipyridyl cation salt (0.52 g, 1.0 mmol) dissolved in methanol (5 ml) was added to the reaction mixture at room temperature. The resulting precipitate was dissolved in DMSO (3 ml) and placed in a sealed jar of anhydrous ether. Red crystals were produced two weeks later under an argon-protected atmosphere. After filtering and drying under vacuum, red needle-shaped crystals of 0.73 g (72.3%) with high quality were obtained. Analysis calculated for C_62_H_102_I_14_N_6_O_4_Pb_4_S_4_: C 19.97, H 2.70, N 2.25%. Found: C 19.80, H 2.82, N 2.25%. IR (cm^−1^): 3291 (*w*), 3108 (*m*), 3035 (*s*), 2931 (*w*), 2958 (*w*), 2857 (*w*), 944 (*w*), 1636 (*m*), 1634 (*s*), 1441 (*m*), 1060 (*s*), 833 (*s*).

## Refinement   

Crystal data, data collection and structure refinement details are summarized in Table 3 ▸. Hydrogen atoms were placed in calculated positions (C—H = 0.93–0.97 Å) and were included in the refinement in the riding-model approximation, with *U*_iso_(H)= 1.2-1.5*U*_eq_(C).

## Supplementary Material

Crystal structure: contains datablock(s) I. DOI: 10.1107/S2056989018016584/ex2016sup1.cif

Structure factors: contains datablock(s) I. DOI: 10.1107/S2056989018016584/ex2016Isup2.hkl

CCDC reference: 1880239

Additional supporting information:  crystallographic information; 3D view; checkCIF report

## Figures and Tables

**Table 1:** Hydrogen-bond geometry (Å, °)

*D*—H⋯*A*	*D*—H	H⋯*A*	*D*⋯*A*	*D*—H⋯*A*
C8—H8⋯I2^i^	0.93	2.94	3.668 (8)	136
C18—H18⋯O1^ii^	0.93	2.30	3.093 (9)	142
C21—H21⋯I7^iii^	0.93	2.95	3.780 (7)	150
C22—H22⋯I2^ii^	0.93	2.86	3.776 (8)	168
C23—H23⋯I1^iv^	0.93	2.85	3.753 (7)	165
C24—H24*B*⋯I5^v^	0.97	2.99	3.940 (10)	166
C30—H30*C*⋯O2^ii^	0.96	2.57	3.517 (10)	169

Symmetry codes: (i) 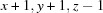; (ii) 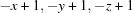; (iii) 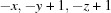; (iv) 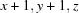; (v) 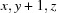.

**Table 2:** Analysis of short ring–ring inter­actions (Å, °) *Cg*(*I*)⋯*Cg*(*J*): ring centroid *I*,*J* (numbered as in Fig. 1 ▸); *Cg*⋯*Cg*: distance between ring centroids; α: dihedral angle between planes *I* and *J*; *CgI*_Perp: perpendicular distance of *Cg*(*I*) on ring J; *CgJ*_Perp: perpendicular distance of *Cg*(*J*) on ring *I*.

*Cg*(*I*)⋯*Cg*(*J*)	*Cg*⋯*Cg*	α	*CgI*_Perp	*CgJ*_Perp
*Cg*(2)⋯*Cg*(3)^vi^	4.796 (4)	27.5 (3)	3.481 (3)	3.970 (3)
*Cg*(3)⋯*Cg*(2)^vi^	4.795 (4)	27.5 (3)	3.970 (3)	3.480 (3)
*Cg*(3)⋯*Cg*(3)^vi^	4.249 (4)	0.0 (4)	3.507 (3)	3.507 (3)

Symmetry code: (vi) 1 − *x*, 2 − *y*, 1 − *z*.

**Table 3:** Experimental details

Crystal data	
Chemical formula	(C_18_H_26_N_2_)_3_[Pb_4_I_14_(C_2_H_6_OS)_2_]·2C_2_H_6_OS
*M*_r_	1864.54
Crystal system, space group	Triclinic, *P* 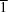
Temperature (K)	296
*a*, *b*, *c* (Å)	11.5011 (10), 14.2262 (13), 16.2969 (14)
α, β, γ (°)	80.305 (1), 78.449 (1), 81.753 (1)
*V* (Å^3^)	2558.5 (4)
*Z*	2
Radiation type	Mo *K*α
μ (mm^−1^)	10.90
Crystal size (mm)	0.55 × 0.50 × 0.09

Data collection	
Diffractometer	Bruker APEX3 CCD area-detector
Absorption correction	Multi-scan (*SADABS*; Bruker, 2017 ▸)
*T*_min_, *T*_max_	0.065, 0.440
No. of measured, independent and observed [*I* > 2σ(*I*)] reflections	24466, 8975, 8219
*R*_int_	0.040
(sin θ/λ)_max_ (Å^−1^)	0.595

Refinement	
*R*[*F* ^2^ > 2σ(*F* ^2^)], *wR*(*F* ^2^), *S*	0.034, 0.099, 1.07
No. of reflections	8975
No. of parameters	432
H-atom treatment	H atoms treated by a mixture of independent and constrained refinement
Δρ_max_, Δρ_min_ (e Å^−3^)	2.47, −1.72

Computer programs: *APEX3* and *SAINT* (Bruker, 2017 ▸), *SHELXTL* (Sheldrick, 2008 ▸).

## supplementary crystallographic information

### Crystal data

**Table:**

(C_18_H_26_N_2_)_3_[Pb_4_I_14_(C_2_H_6_OS)_2_]·2C_2_H_6_OS	*Z* = 2
*M**_r_* = 1864.54	*F*(000) = 1682
Triclinic, *P*1	*D*_x_ = 2.419 Mg m^−^^3^
Hall symbol: -P 1	Mo *K*α radiation, λ = 0.71073 Å
*a* = 11.5011 (10) Å	Cell parameters from 29882 reflections
*b* = 14.2262 (13) Å	θ = 1.8–25°
*c* = 16.2969 (14) Å	µ = 10.90 mm^−^^1^
α = 80.305 (1)°	*T* = 296 K
β = 78.449 (1)°	Needle, red
γ = 81.753 (1)°	0.55 × 0.50 × 0.09 mm
*V* = 2558.5 (4) Å^3^	

### Data collection

**Table:**

Bruker APEX3 CCD area-detector diffractometer	8975 independent reflections
Radiation source: fine-focus sealed tube	8219 reflections with *I* > 2σ(*I*)
Graphite monochromator	*R*_int_ = 0.040
φ and ω scans	θ_max_ = 25.0°, θ_min_ = 1.8°
Absorption correction: multi-scan (SADABS; Bruker, 2017)	*h* = −13→13
*T*_min_ = 0.065, *T*_max_ = 0.440	*k* = −16→16
24466 measured reflections	*l* = −19→19

### Refinement

**Table:**

Refinement on *F*^2^	Secondary atom site location: difference Fourier map
Least-squares matrix: full	Hydrogen site location: inferred from neighbouring sites
*R*[*F*^2^ > 2σ(*F*^2^)] = 0.034	H atoms treated by a mixture of independent and constrained refinement
*wR*(*F*^2^) = 0.099	*w* = 1/[σ^2^(*F*_o_^2^) + (0.0562*P*)^2^ + 3.2208*P*] where *P* = (*F*_o_^2^ + 2*F*_c_^2^)/3
*S* = 1.07	(Δ/σ)_max_ = 0.059
8975 reflections	Δρ_max_ = 2.47 e Å^−^^3^
432 parameters	Δρ_min_ = −1.72 e Å^−^^3^
0 restraints	Extinction correction: SHELXTL (Bruker, 2017), Fc^*^=kFc[1+0.001xFc^2^λ^3^/sin(2θ)]^-1/4^
Primary atom site location: structure-invariant direct methods	Extinction coefficient: 0.00081 (6)

### Special details

**Table:**

Geometry. All esds (except the esd in the dihedral angle between two l.s. planes) are estimated using the full covariance matrix. The cell esds are taken into account individually in the estimation of esds in distances, angles and torsion angles; correlations between esds in cell parameters are only used when they are defined by crystal symmetry. An approximate (isotropic) treatment of cell esds is used for estimating esds involving l.s. planes.
Refinement. Refinement of F^2^ against ALL reflections. The weighted R-factor wR and goodness of fit S are based on F^2^, conventional R-factors R are based on F, with F set to zero for negative F^2^. The threshold expression of F^2^ > 2sigma(F^2^) is used only for calculating R-factors(gt) etc. and is not relevant to the choice of reflections for refinement. R-factors based on F^2^ are statistically about twice as large as those based on F, and R- factors based on ALL data will be even larger.

### Fractional atomic coordinates and isotropic or equivalent isotropic displacement parameters (Å^2^)

**Table:**

	*x*	*y*	*z*	*U*_iso_*/*U*_eq_	
Pb1	−0.019011 (19)	0.115698 (16)	0.614407 (14)	0.02456 (9)	
Pb2	0.056844 (19)	0.234124 (16)	0.300143 (15)	0.02475 (9)	
I3	−0.12457 (3)	0.27930 (3)	0.48471 (3)	0.02923 (11)	
I4	−0.07737 (4)	0.39067 (3)	0.18256 (3)	0.04282 (14)	
I5	0.27864 (4)	0.19949 (4)	0.16274 (3)	0.04566 (14)	
I6	0.15180 (4)	0.24586 (3)	0.65461 (3)	0.04339 (14)	
I7	−0.22112 (4)	0.16826 (4)	0.75974 (3)	0.04122 (13)	
C7	0.9915 (6)	0.9485 (5)	0.0111 (4)	0.0338 (15)	
N2	0.4596 (5)	0.6917 (4)	0.7240 (4)	0.0363 (13)	
C1	0.5880 (9)	0.6807 (9)	0.1101 (8)	0.087 (3)	
H1A	0.5779	0.7078	0.0535	0.130*	
H1B	0.5298	0.7132	0.1499	0.130*	
H1C	0.5775	0.6137	0.1193	0.130*	
C2	0.7139 (8)	0.6923 (7)	0.1220 (7)	0.068 (3)	
H2A	0.7239	0.7601	0.1120	0.082*	
H2B	0.7218	0.6681	0.1801	0.082*	
C3	0.8105 (7)	0.6412 (5)	0.0644 (5)	0.050 (2)	
H3A	0.8006	0.6641	0.0064	0.060*	
H3B	0.8012	0.5733	0.0756	0.060*	
C4	0.9363 (7)	0.6536 (5)	0.0728 (5)	0.0496 (19)	
H4A	0.9479	0.6299	0.1303	0.059*	
H4B	0.9935	0.6161	0.0352	0.059*	
N1	0.9583 (5)	0.7570 (4)	0.0515 (4)	0.0368 (13)	
C5	0.9825 (8)	0.8037 (6)	0.1095 (5)	0.055 (2)	
H5	0.9881	0.7715	0.1635	0.066*	
C6	0.9992 (8)	0.8996 (6)	0.0897 (5)	0.055 (2)	
H6	1.0160	0.9314	0.1306	0.067*	
C9	0.9507 (8)	0.8038 (7)	−0.0262 (5)	0.060 (2)	
H9	0.9343	0.7710	−0.0666	0.072*	
C8	0.9665 (9)	0.8976 (7)	−0.0468 (5)	0.063 (3)	
H8	0.9604	0.9284	−0.1011	0.076*	
C10	0.2978 (9)	0.6823 (9)	1.0341 (6)	0.084 (3)	
H10A	0.2572	0.6397	1.0793	0.127*	
H10B	0.2407	0.7316	1.0127	0.127*	
H10C	0.3547	0.7110	1.0548	0.127*	
C11	0.3623 (9)	0.6262 (7)	0.9635 (6)	0.067 (3)	
H11A	0.3150	0.5765	0.9594	0.081*	
H11B	0.4382	0.5951	0.9771	0.081*	
C12	0.3845 (7)	0.6903 (6)	0.8783 (5)	0.0481 (19)	
H12A	0.3091	0.7237	0.8656	0.058*	
H12B	0.4353	0.7380	0.8812	0.058*	
C13	0.4431 (7)	0.6323 (5)	0.8086 (5)	0.0446 (18)	
H13A	0.5203	0.6018	0.8201	0.054*	
H13B	0.3944	0.5821	0.8083	0.054*	
C14	0.3627 (6)	0.7293 (5)	0.6908 (5)	0.0381 (16)	
H14	0.2872	0.7185	0.7213	0.046*	
C15	0.3738 (5)	0.7837 (5)	0.6123 (5)	0.0393 (17)	
H15	0.3059	0.8096	0.5899	0.047*	
C16	0.4857 (5)	0.8001 (5)	0.5664 (5)	0.0310 (15)	
C19	0.4996 (5)	0.8580 (5)	0.4816 (5)	0.0330 (16)	
C23	0.5989 (6)	0.9090 (5)	0.4521 (5)	0.0392 (16)	
H23	0.6563	0.9058	0.4856	0.047*	
C22	0.6111 (6)	0.9628 (6)	0.3748 (5)	0.0468 (19)	
H22	0.6775	0.9962	0.3555	0.056*	
N3	0.5285 (5)	0.9690 (4)	0.3252 (4)	0.0419 (15)	
C24	0.5471 (8)	1.0264 (7)	0.2402 (6)	0.064 (3)	
H24A	0.6019	1.0729	0.2383	0.077*	
H24B	0.4717	1.0613	0.2290	0.077*	
C25	0.5966 (9)	0.9636 (9)	0.1732 (6)	0.076 (3)	
H25A	0.5386	0.9216	0.1703	0.091*	
H25B	0.6685	0.9242	0.1864	0.091*	
C26	0.6271 (12)	1.0332 (11)	0.0828 (8)	0.112 (5)	
H26A	0.5573	1.0779	0.0735	0.134*	
H26B	0.6913	1.0699	0.0842	0.134*	
C27	0.6594 (15)	0.9805 (13)	0.0197 (10)	0.140 (6)	
H27A	0.7293	0.9371	0.0286	0.209*	
H27B	0.6767	1.0219	−0.0331	0.209*	
H27C	0.5955	0.9445	0.0183	0.209*	
C21	0.4319 (6)	0.9209 (5)	0.3527 (5)	0.0430 (19)	
H21	0.3755	0.9253	0.3181	0.052*	
C20	0.4156 (5)	0.8659 (5)	0.4301 (5)	0.0363 (16)	
H20	0.3480	0.8338	0.4483	0.044*	
C17	0.5853 (5)	0.7597 (5)	0.6030 (5)	0.0378 (16)	
H17	0.6619	0.7693	0.5740	0.045*	
C18	0.5692 (6)	0.7060 (5)	0.6819 (5)	0.0372 (16)	
H18	0.6353	0.6794	0.7062	0.045*	
S1	0.09717 (14)	0.45421 (12)	0.35969 (11)	0.0341 (4)	
O1	0.1616 (4)	0.3631 (3)	0.3269 (3)	0.0348 (11)	
C30	0.1581 (7)	0.4585 (6)	0.4499 (5)	0.058 (2)	
H30A	0.1352	0.4060	0.4928	0.087*	
H30B	0.1285	0.5181	0.4710	0.087*	
H30C	0.2436	0.4537	0.4349	0.087*	
C31	0.1638 (9)	0.5487 (6)	0.2894 (7)	0.078 (3)	
H31A	0.2470	0.5437	0.2926	0.117*	
H31B	0.1248	0.6092	0.3047	0.117*	
H31C	0.1559	0.5445	0.2326	0.117*	
S2	0.44397 (18)	0.46100 (15)	0.66133 (14)	0.0493 (5)	
O2	0.5347 (5)	0.5287 (4)	0.6236 (4)	0.0671 (18)	
C28	0.3245 (8)	0.4909 (6)	0.6066 (6)	0.062 (2)	
H28A	0.2831	0.5519	0.6188	0.092*	
H28B	0.2707	0.4426	0.6240	0.092*	
H28C	0.3543	0.4945	0.5469	0.092*	
C29	0.4998 (9)	0.3496 (7)	0.6250 (9)	0.091 (4)	
H29A	0.4961	0.3549	0.5660	0.136*	
H29B	0.4523	0.3008	0.6566	0.136*	
H29C	0.5812	0.3327	0.6327	0.136*	
I2	0.10647 (3)	−0.07348 (3)	0.72885 (3)	0.02993 (12)	
I1	−0.16306 (3)	−0.05420 (3)	0.56721 (3)	0.02845 (12)	

### Atomic displacement parameters (Å^2^)

**Table:**

	*U*^11^	*U*^22^	*U*^33^	*U*^12^	*U*^13^	*U*^23^
Pb1	0.02601 (14)	0.02141 (14)	0.02844 (15)	−0.00252 (10)	−0.00949 (10)	−0.00442 (10)
Pb2	0.02510 (14)	0.02060 (14)	0.03053 (15)	−0.00131 (10)	−0.00997 (10)	−0.00449 (10)
I3	0.0275 (2)	0.0228 (2)	0.0371 (2)	−0.00003 (16)	−0.00946 (17)	−0.00164 (18)
I4	0.0412 (3)	0.0305 (3)	0.0544 (3)	−0.0011 (2)	−0.0192 (2)	0.0108 (2)
I5	0.0392 (3)	0.0600 (3)	0.0366 (3)	0.0084 (2)	−0.0076 (2)	−0.0147 (2)
I6	0.0441 (3)	0.0374 (3)	0.0561 (3)	−0.0159 (2)	−0.0173 (2)	−0.0080 (2)
I7	0.0369 (2)	0.0492 (3)	0.0397 (3)	−0.0072 (2)	−0.00319 (19)	−0.0148 (2)
C7	0.029 (3)	0.044 (4)	0.032 (4)	−0.002 (3)	−0.010 (3)	−0.011 (3)
N2	0.032 (3)	0.036 (3)	0.043 (3)	0.002 (2)	−0.005 (2)	−0.021 (3)
C1	0.061 (6)	0.100 (9)	0.100 (9)	−0.029 (6)	−0.022 (6)	0.008 (7)
C2	0.058 (5)	0.063 (6)	0.086 (7)	−0.017 (5)	−0.012 (5)	−0.009 (5)
C3	0.072 (5)	0.026 (4)	0.056 (5)	−0.014 (4)	−0.031 (4)	0.009 (3)
C4	0.063 (5)	0.037 (4)	0.053 (5)	−0.004 (4)	−0.027 (4)	0.001 (4)
N1	0.043 (3)	0.032 (3)	0.040 (3)	−0.003 (3)	−0.016 (3)	−0.008 (3)
C5	0.088 (6)	0.041 (5)	0.039 (4)	0.013 (4)	−0.037 (4)	0.000 (4)
C6	0.093 (6)	0.043 (5)	0.045 (5)	0.006 (4)	−0.046 (4)	−0.018 (4)
C9	0.091 (7)	0.071 (6)	0.031 (4)	−0.046 (5)	−0.013 (4)	−0.008 (4)
C8	0.106 (7)	0.069 (6)	0.028 (4)	−0.053 (6)	−0.019 (4)	0.001 (4)
C10	0.072 (7)	0.132 (11)	0.045 (5)	−0.002 (6)	−0.003 (5)	−0.018 (6)
C11	0.074 (6)	0.071 (7)	0.055 (6)	−0.006 (5)	−0.013 (5)	−0.007 (5)
C12	0.036 (4)	0.059 (5)	0.051 (5)	−0.002 (4)	−0.005 (3)	−0.020 (4)
C13	0.049 (4)	0.040 (4)	0.047 (5)	0.004 (3)	−0.012 (3)	−0.014 (4)
C14	0.029 (3)	0.040 (4)	0.046 (4)	−0.003 (3)	−0.002 (3)	−0.015 (3)
C15	0.018 (3)	0.046 (4)	0.058 (5)	0.001 (3)	−0.007 (3)	−0.024 (4)
C16	0.025 (3)	0.029 (3)	0.043 (4)	0.000 (3)	−0.007 (3)	−0.019 (3)
C19	0.020 (3)	0.033 (4)	0.050 (4)	0.002 (3)	−0.004 (3)	−0.024 (3)
C23	0.026 (3)	0.047 (4)	0.047 (4)	−0.010 (3)	−0.010 (3)	−0.007 (4)
C22	0.027 (3)	0.049 (5)	0.067 (5)	−0.006 (3)	−0.008 (3)	−0.014 (4)
N3	0.029 (3)	0.044 (4)	0.048 (4)	0.008 (3)	−0.005 (3)	−0.008 (3)
C24	0.040 (4)	0.082 (7)	0.063 (6)	0.016 (4)	−0.015 (4)	−0.002 (5)
C25	0.072 (6)	0.108 (9)	0.049 (6)	−0.012 (6)	−0.015 (5)	−0.011 (6)
C26	0.108 (10)	0.143 (12)	0.092 (9)	0.056 (9)	−0.055 (8)	−0.052 (9)
C27	0.136 (13)	0.164 (16)	0.130 (14)	0.020 (11)	−0.042 (11)	−0.062 (12)
C21	0.020 (3)	0.043 (4)	0.072 (6)	0.007 (3)	−0.017 (3)	−0.023 (4)
C20	0.023 (3)	0.040 (4)	0.050 (4)	−0.001 (3)	−0.008 (3)	−0.017 (4)
C17	0.021 (3)	0.038 (4)	0.058 (5)	0.000 (3)	−0.011 (3)	−0.015 (4)
C18	0.029 (3)	0.033 (4)	0.055 (5)	0.004 (3)	−0.014 (3)	−0.020 (3)
S1	0.0289 (8)	0.0265 (8)	0.0481 (10)	−0.0021 (6)	−0.0051 (7)	−0.0117 (7)
O1	0.026 (2)	0.031 (2)	0.052 (3)	−0.0029 (18)	−0.007 (2)	−0.021 (2)
C30	0.056 (5)	0.064 (6)	0.065 (6)	−0.002 (4)	−0.013 (4)	−0.040 (5)
C31	0.065 (6)	0.039 (5)	0.123 (9)	−0.012 (4)	−0.009 (6)	0.006 (5)
S2	0.0485 (11)	0.0429 (11)	0.0629 (13)	−0.0001 (9)	−0.0184 (9)	−0.0197 (10)
O2	0.049 (3)	0.050 (4)	0.113 (5)	−0.012 (3)	−0.015 (3)	−0.037 (4)
C28	0.066 (5)	0.037 (5)	0.088 (7)	−0.012 (4)	−0.040 (5)	0.009 (4)
C29	0.060 (6)	0.050 (6)	0.168 (12)	0.004 (5)	−0.011 (7)	−0.049 (7)
I2	0.0277 (2)	0.0258 (2)	0.0380 (2)	−0.00431 (17)	−0.00994 (17)	−0.00347 (18)
I1	0.0271 (2)	0.0254 (2)	0.0363 (2)	−0.00027 (16)	−0.01434 (17)	−0.00608 (18)

### Geometric parameters (Å, º)

**Table:**

Pb1—I7	3.0765 (5)	C14—H14	0.9300
Pb1—I6	3.1121 (5)	C15—C16	1.383 (9)
Pb1—I3	3.1493 (5)	C15—H15	0.9300
Pb1—I2	3.3282 (5)	C16—C17	1.403 (9)
Pb1—I1	3.3858 (5)	C16—C19	1.476 (10)
Pb2—O1	2.473 (4)	C19—C20	1.384 (9)
Pb2—I5	3.0802 (5)	C19—C23	1.400 (9)
Pb2—I4	3.1266 (5)	C23—C22	1.351 (11)
Pb2—I2^i^	3.3053 (5)	C23—H23	0.9300
Pb2—I1^i^	3.3187 (5)	C22—N3	1.350 (10)
Pb2—I3	3.4010 (5)	C22—H22	0.9300
C7—C6	1.364 (10)	N3—C21	1.348 (9)
C7—C8	1.378 (10)	N3—C24	1.479 (11)
C7—C7^ii^	1.481 (14)	C24—C25	1.499 (13)
N2—C18	1.335 (9)	C24—H24A	0.9700
N2—C14	1.340 (9)	C24—H24B	0.9700
N2—C13	1.483 (10)	C25—C26	1.636 (17)
C1—C2	1.534 (13)	C25—H25A	0.9700
C1—H1A	0.9600	C25—H25B	0.9700
C1—H1B	0.9600	C26—C27	1.336 (17)
C1—H1C	0.9600	C26—H26A	0.9700
C2—C3	1.491 (12)	C26—H26B	0.9700
C2—H2A	0.9700	C27—H27A	0.9600
C2—H2B	0.9700	C27—H27B	0.9600
C3—C4	1.518 (11)	C27—H27C	0.9600
C3—H3A	0.9700	C21—C20	1.361 (11)
C3—H3B	0.9700	C21—H21	0.9300
C4—N1	1.499 (9)	C20—H20	0.9300
C4—H4A	0.9700	C17—C18	1.373 (10)
C4—H4B	0.9700	C17—H17	0.9300
N1—C5	1.336 (9)	C18—H18	0.9300
N1—C9	1.341 (10)	S1—O1	1.522 (4)
C5—C6	1.380 (11)	S1—C30	1.763 (8)
C5—H5	0.9300	S1—C31	1.770 (9)
C6—H6	0.9300	C30—H30A	0.9600
C9—C8	1.349 (12)	C30—H30B	0.9600
C9—H9	0.9300	C30—H30C	0.9600
C8—H8	0.9300	C31—H31A	0.9600
C10—C11	1.520 (13)	C31—H31B	0.9600
C10—H10A	0.9600	C31—H31C	0.9600
C10—H10B	0.9600	S2—O2	1.492 (6)
C10—H10C	0.9600	S2—C28	1.749 (8)
C11—C12	1.524 (12)	S2—C29	1.774 (9)
C11—H11A	0.9700	C28—H28A	0.9600
C11—H11B	0.9700	C28—H28B	0.9600
C12—C13	1.508 (10)	C28—H28C	0.9600
C12—H12A	0.9700	C29—H29A	0.9600
C12—H12B	0.9700	C29—H29B	0.9600
C13—H13A	0.9700	C29—H29C	0.9600
C13—H13B	0.9700	I2—Pb2^i^	3.3053 (5)
C14—C15	1.371 (10)	I1—Pb2^i^	3.3187 (5)
			
I7—Pb1—I6	93.528 (15)	C12—C13—H13B	109.1
I7—Pb1—I3	91.655 (15)	H13A—C13—H13B	107.8
I6—Pb1—I3	93.038 (15)	N2—C14—C15	120.7 (6)
I7—Pb1—I2	95.153 (14)	N2—C14—H14	119.7
I6—Pb1—I2	90.519 (14)	C15—C14—H14	119.7
I3—Pb1—I2	172.112 (13)	C14—C15—C16	120.2 (7)
I7—Pb1—I1	93.512 (14)	C14—C15—H15	119.9
I6—Pb1—I1	170.159 (14)	C16—C15—H15	119.9
I3—Pb1—I1	93.622 (13)	C15—C16—C17	117.8 (7)
I2—Pb1—I1	82.007 (13)	C15—C16—C19	121.0 (6)
O1—Pb2—I5	84.47 (10)	C17—C16—C19	121.2 (6)
O1—Pb2—I4	87.45 (11)	C20—C19—C23	118.0 (7)
I5—Pb2—I4	94.693 (16)	C20—C19—C16	122.1 (6)
O1—Pb2—I2^i^	174.71 (9)	C23—C19—C16	119.9 (6)
I5—Pb2—I2^i^	100.234 (14)	C22—C23—C19	119.9 (7)
I4—Pb2—I2^i^	89.734 (15)	C22—C23—H23	120.0
O1—Pb2—I1^i^	99.08 (11)	C19—C23—H23	120.0
I5—Pb2—I1^i^	90.803 (14)	N3—C22—C23	121.1 (7)
I4—Pb2—I1^i^	171.859 (14)	N3—C22—H22	119.5
I2^i^—Pb2—I1^i^	83.372 (13)	C23—C22—H22	119.5
O1—Pb2—I3	82.78 (10)	C21—N3—C22	119.9 (7)
I5—Pb2—I3	162.883 (14)	C21—N3—C24	120.8 (7)
I4—Pb2—I3	96.095 (14)	C22—N3—C24	119.3 (7)
I2^i^—Pb2—I3	93.076 (12)	N3—C24—C25	111.0 (8)
I1^i^—Pb2—I3	80.016 (12)	N3—C24—H24A	109.4
Pb1—I3—Pb2	100.953 (13)	C25—C24—H24A	109.4
C6—C7—C8	116.9 (7)	N3—C24—H24B	109.4
C6—C7—C7^ii^	121.8 (7)	C25—C24—H24B	109.4
C8—C7—C7^ii^	121.3 (8)	H24A—C24—H24B	108.0
C18—N2—C14	121.1 (7)	C24—C25—C26	107.9 (9)
C18—N2—C13	120.3 (6)	C24—C25—H25A	110.1
C14—N2—C13	118.6 (6)	C26—C25—H25A	110.1
C2—C1—H1A	109.5	C24—C25—H25B	110.1
C2—C1—H1B	109.5	C26—C25—H25B	110.1
H1A—C1—H1B	109.5	H25A—C25—H25B	108.4
C2—C1—H1C	109.5	C27—C26—C25	110.1 (14)
H1A—C1—H1C	109.5	C27—C26—H26A	109.6
H1B—C1—H1C	109.5	C25—C26—H26A	109.6
C3—C2—C1	113.3 (9)	C27—C26—H26B	109.7
C3—C2—H2A	108.9	C25—C26—H26B	109.6
C1—C2—H2A	108.9	H26A—C26—H26B	108.1
C3—C2—H2B	108.9	C26—C27—H27A	109.5
C1—C2—H2B	108.9	C26—C27—H27B	109.5
H2A—C2—H2B	107.7	H27A—C27—H27B	109.5
C2—C3—C4	114.6 (7)	C26—C27—H27C	109.5
C2—C3—H3A	108.6	H27A—C27—H27C	109.5
C4—C3—H3A	108.6	H27B—C27—H27C	109.5
C2—C3—H3B	108.6	N3—C21—C20	121.1 (6)
C4—C3—H3B	108.6	N3—C21—H21	119.4
H3A—C3—H3B	107.6	C20—C21—H21	119.4
N1—C4—C3	111.1 (6)	C21—C20—C19	119.9 (6)
N1—C4—H4A	109.4	C21—C20—H20	120.1
C3—C4—H4A	109.4	C19—C20—H20	120.1
N1—C4—H4B	109.4	C18—C17—C16	119.7 (6)
C3—C4—H4B	109.4	C18—C17—H17	120.1
H4A—C4—H4B	108.0	C16—C17—H17	120.1
C5—N1—C9	119.6 (7)	N2—C18—C17	120.6 (6)
C5—N1—C4	120.8 (6)	N2—C18—H18	119.7
C9—N1—C4	119.6 (6)	C17—C18—H18	119.7
N1—C5—C6	120.2 (7)	O1—S1—C30	104.1 (3)
N1—C5—H5	119.9	O1—S1—C31	104.7 (4)
C6—C5—H5	119.9	C30—S1—C31	99.9 (5)
C7—C6—C5	121.0 (7)	S1—O1—Pb2	123.4 (2)
C7—C6—H6	119.5	S1—C30—H30A	109.5
C5—C6—H6	119.5	S1—C30—H30B	109.5
N1—C9—C8	121.2 (7)	H30A—C30—H30B	109.5
N1—C9—H9	119.4	S1—C30—H30C	109.5
C8—C9—H9	119.4	H30A—C30—H30C	109.5
C9—C8—C7	121.1 (8)	H30B—C30—H30C	109.5
C9—C8—H8	119.4	S1—C31—H31A	109.5
C7—C8—H8	119.5	S1—C31—H31B	109.5
C11—C10—H10A	109.5	H31A—C31—H31B	109.5
C11—C10—H10B	109.5	S1—C31—H31C	109.5
H10A—C10—H10B	109.5	H31A—C31—H31C	109.5
C11—C10—H10C	109.5	H31B—C31—H31C	109.5
H10A—C10—H10C	109.5	O2—S2—C28	108.1 (4)
H10B—C10—H10C	109.5	O2—S2—C29	107.1 (4)
C10—C11—C12	112.1 (8)	C28—S2—C29	98.0 (5)
C10—C11—H11A	109.2	S2—C28—H28A	109.5
C12—C11—H11A	109.2	S2—C28—H28B	109.5
C10—C11—H11B	109.2	H28A—C28—H28B	109.5
C12—C11—H11B	109.2	S2—C28—H28C	109.5
H11A—C11—H11B	107.9	H28A—C28—H28C	109.5
C13—C12—C11	111.1 (7)	H28B—C28—H28C	109.5
C13—C12—H12A	109.4	S2—C29—H29A	109.5
C11—C12—H12A	109.4	S2—C29—H29B	109.5
C13—C12—H12B	109.4	H29A—C29—H29B	109.5
C11—C12—H12B	109.4	S2—C29—H29C	109.5
H12A—C12—H12B	108.0	H29A—C29—H29C	109.5
N2—C13—C12	112.5 (6)	H29B—C29—H29C	109.5
N2—C13—H13A	109.1	Pb2^i^—I2—Pb1	97.673 (13)
C12—C13—H13A	109.1	Pb2^i^—I1—Pb1	96.290 (13)
N2—C13—H13B	109.1		
			
I7—Pb1—I3—Pb2	−173.613 (13)	C17—C16—C19—C23	−28.4 (9)
I6—Pb1—I3—Pb2	92.765 (15)	C20—C19—C23—C22	−0.9 (10)
I2—Pb1—I3—Pb2	−23.93 (9)	C16—C19—C23—C22	−179.3 (6)
I1—Pb1—I3—Pb2	−79.987 (13)	C19—C23—C22—N3	0.4 (11)
O1—Pb2—I3—Pb1	−98.01 (11)	C23—C22—N3—C21	0.0 (11)
I5—Pb2—I3—Pb1	−55.87 (5)	C23—C22—N3—C24	−178.2 (7)
I4—Pb2—I3—Pb1	175.355 (13)	C21—N3—C24—C25	−79.3 (9)
I2^i^—Pb2—I3—Pb1	85.294 (14)	C22—N3—C24—C25	98.9 (9)
I1^i^—Pb2—I3—Pb1	2.580 (11)	N3—C24—C25—C26	−174.4 (8)
C1—C2—C3—C4	178.3 (8)	C24—C25—C26—C27	−173.3 (11)
C2—C3—C4—N1	−61.5 (9)	C22—N3—C21—C20	0.2 (10)
C3—C4—N1—C5	117.3 (8)	C24—N3—C21—C20	178.4 (7)
C3—C4—N1—C9	−61.1 (10)	N3—C21—C20—C19	−0.7 (10)
C9—N1—C5—C6	0.2 (12)	C23—C19—C20—C21	1.1 (9)
C4—N1—C5—C6	−178.2 (8)	C16—C19—C20—C21	179.4 (6)
C8—C7—C6—C5	−0.1 (13)	C15—C16—C17—C18	0.0 (9)
C7^ii^—C7—C6—C5	179.7 (8)	C19—C16—C17—C18	−179.8 (6)
N1—C5—C6—C7	0.0 (14)	C14—N2—C18—C17	−0.2 (9)
C5—N1—C9—C8	−0.4 (13)	C13—N2—C18—C17	179.1 (6)
C4—N1—C9—C8	178.1 (8)	C16—C17—C18—N2	0.2 (10)
N1—C9—C8—C7	0.3 (15)	C30—S1—O1—Pb2	126.1 (4)
C6—C7—C8—C9	−0.1 (14)	C31—S1—O1—Pb2	−129.5 (5)
C7^ii^—C7—C8—C9	−179.8 (9)	I5—Pb2—O1—S1	150.1 (3)
C10—C11—C12—C13	−177.2 (8)	I4—Pb2—O1—S1	55.2 (3)
C18—N2—C13—C12	112.7 (7)	I2^i^—Pb2—O1—S1	−2.7 (15)
C14—N2—C13—C12	−68.0 (8)	I1^i^—Pb2—O1—S1	−119.9 (3)
C11—C12—C13—N2	176.7 (7)	I3—Pb2—O1—S1	−41.3 (3)
C18—N2—C14—C15	0.1 (10)	I7—Pb1—I2—Pb2^i^	86.695 (15)
C13—N2—C14—C15	−179.2 (6)	I6—Pb1—I2—Pb2^i^	−179.716 (13)
N2—C14—C15—C16	0.1 (10)	I3—Pb1—I2—Pb2^i^	−62.87 (9)
C14—C15—C16—C17	−0.2 (9)	I1—Pb1—I2—Pb2^i^	−6.140 (11)
C14—C15—C16—C19	179.6 (6)	I7—Pb1—I1—Pb2^i^	−88.621 (15)
C15—C16—C19—C20	−26.5 (9)	I6—Pb1—I1—Pb2^i^	46.99 (9)
C17—C16—C19—C20	153.3 (6)	I3—Pb1—I1—Pb2^i^	179.495 (10)
C15—C16—C19—C23	151.8 (6)	I2—Pb1—I1—Pb2^i^	6.097 (11)

Symmetry codes: (i) −*x*, −*y*, −*z*+1; (ii) −*x*+2, −*y*+2, −*z*.

### Hydrogen-bond geometry (Å, º)

**Table:**

*D*—H···*A*	*D*—H	H···*A*	*D*···*A*	*D*—H···*A*
C8—H8···I2^iii^	0.93	2.94	3.668 (8)	136
C18—H18···O1^iv^	0.93	2.30	3.093 (9)	142
C21—H21···I7^v^	0.93	2.95	3.780 (7)	150
C22—H22···I2^iv^	0.93	2.86	3.776 (8)	168
C23—H23···I1^vi^	0.93	2.85	3.753 (7)	165
C24—H24*B*···I5^vii^	0.97	2.99	3.940 (10)	166
C30—H30*C*···O2^iv^	0.96	2.57	3.517 (10)	169

Symmetry codes: (iii) *x*+1, *y*+1, *z*−1; (iv) −*x*+1, −*y*+1, −*z*+1; (v) −*x*, −*y*+1, −*z*+1; (vi) *x*+1, *y*+1, *z*; (vii) *x*, *y*+1, *z*.

### The fractional coordinates of Cg(I)

**Table:**

Cg(I)	x	y	z
Cg(1)	0.9749 (3)	0.8517 (2)	0.03147 (19)
Cg(2)	0.4727 (2)	0.7451 (2)	0.6464 (2)
Cg(3)	0.5142 (2)	0.9143 (2)	0.4028 (2)
